# Supplementary material for: An integrative proteomics method identifies a regulator of translation during stem cell maintenance and differentiation
Source: Nat Commun. 2021 Nov 12;12:6558. doi: 10.1038/s41467-021-26879-4 (PMC8590018; doi:10.1038/s41467-021-26879-4)
Supplement: Supplementary file 1 — Supplementary Information [file 41467_2021_26879_MOESM1_ESM.pdf]

An integrative proteomics method identifies a regulator of translation during stem cell maintenance and differentiation

Pierre Sabatier, Christian M. Beusch, Amir A. Saei, Mike Aoun, Noah Moruzzi, Ana Coelho, Niels Leijten, Magnus Nordenskjöld, Patrick Micke, Diana Maltseva, Alexander G. Tonevitsky, Vincent Millischer, J. Carlos Villaescusa, Sandeep Kadekar, Massimiliano Gaetani, Kamilya Altynbekova, Alexander Kel, Per-Olof Berggren, Oscar Simonson, Karl-Henrik Grinnemo, Rikard Holmdahl, Sergey Rodin*, Roman A. Zubarev*

*Corresponding authors. Emails: sergey.rodin@surgsci.uu.se; roman.zubarev@ki.se

Supplementary Figures 1-8
Supplementary Tables 1, 2 and 3

Supplementary information

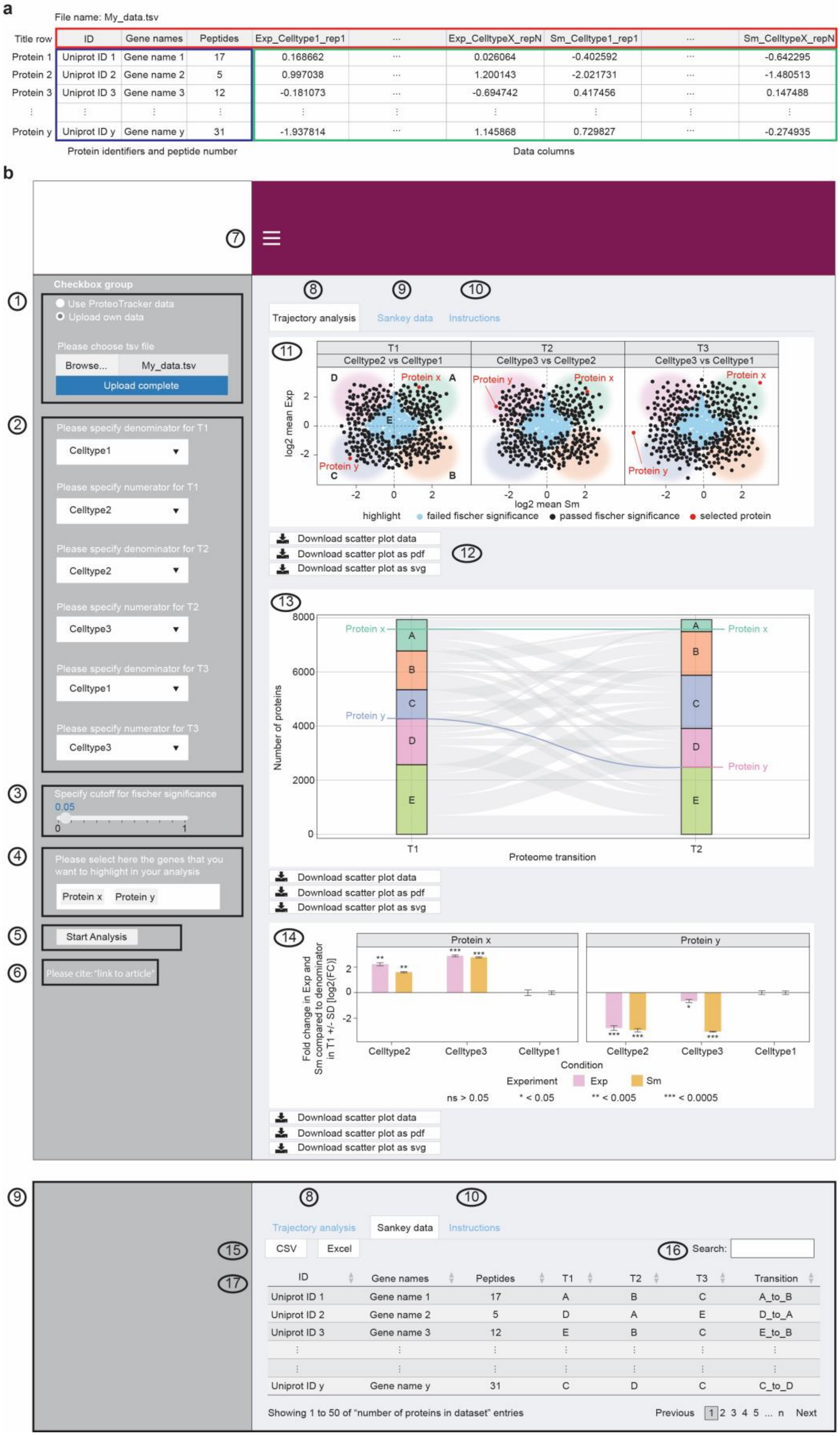
a
File name: My_data.tsv
Title row
ID
Gene names
Peptides
Exp_Celltype1_rep1
Exp_CelltypeX_repN
Sm_Celltype1_rep1
Sm_CelltypeX_repN
Protein 1
Uniprot ID 1
Gene name 1
17
0.168662
0.026064
-0.402592
-0.642295
Protein 2
Uniprot ID 2
Gene name 2
5
0.997038
1.200143
-2.021731
-1.480513
Protein 3
Uniprot ID 3
Gene name 3
12
-0.181073
-0.694742
0.417456
0.147488
Protein y
Uniprot ID y
Gene name y
31
-1.937814
1.145868
0.729827
-0.274935
Protein identifiers and peptide number
Data columns
b
Checkbox group
Use ProteoTracker data
Upload own data
Please choose tsv file
Browse...
My_data.tsv
Upload complete
Please specify denominator for T1
Celltype1
Please specify numerator for T1
Celltype2
Please specify denominator for T2
Celltype2
Please specify numerator for T2
Celltype3
Please specify denominator for T3
Celltype1
Please specify numerator for T3
Celltype3
Specify cutoff for fischer significance
0.05
Please select here the genes that you want to highlight in your analysis
Protein x
Protein y
Start Analysis
Please cite: "link to article"
Trajectory analysis
Sankey data
Instructions
T1
Celltype2 vs Celltype1
T2
Celltype3 vs Celltype2
T3
Celltype3 vs Celltype1
log2 mean Exp
log2 mean Sm
highlight
failed fischer significance
passed fischer significance
selected protein
Download scatter plot data
Download scatter plot as pdf
Download scatter plot as svg
Number of proteins
Proteome transition
Fold change in Exp and Sm compared to denominator in T1 +/- SD [log2(FC)]
Condition
Experiment
Exp
Sm
ns > 0.05
* < 0.05
** < 0.005
*** < 0.0005
CSV
Excel
Search:
ID
Gene names
Peptides
T1
T2
T3
Transition
Uniprot ID 1
Gene name 1
17
A
B
C
A_to_B
Uniprot ID 2
Gene name 2
5
D
A
E
D_to_A
Uniprot ID 3
Gene name 3
12
E
B
C
E_to_B
Uniprot ID y
Gene name y
31
C
D
C
C_to_D
Showing 1 to 50 of "number of proteins in dataset" entries
Previous
Next

Supplementary Figure 1. Overview of ProteoTracker web interface. (a) Layout of a typical .tsv file containing a proteomics dataset that can be submitted to the interface for analysis. Data columns names should be presented as described here with Exp and Sm being the measured parameters as described in Figure 1. Then follows the name of the condition without spacing and finally the replicate number. The parameter, condition and replicate identifiers are to be separated by a semicolon as shown here. Any number of conditions and replicates can be included in the .tsv file. Only Exp_ and Sm_ need to be strictly kept for the naming. Any condition name and replicate name can be included.

(b) Step by step description of the web interface.

1) The user can choose to upload is own data in the format described above or work with the ProteoTracker dataset.

2) The different comparisons can be selected it with maximum 3 conditions at a time.

3) The user can specify the Fisher score cutoff calculated for T1 and T2.

4) The user can select proteins present in the datasets that will appear on the analysis, the name used are the ones entered in the “Gene names” column of the data file.

5) Start button of the analysis.

6) Link to the article.

7) Hide or show the checkbox group.

8) Trajectory analysis default window (the window currently represented on the scheme).

9) The user can visualize the Sankey data (The window is the one presented below the Trajectory analysis window).

10) Instruction window which displays the figure and caption.

11) Two-dimensional plots of Exp and Sm fold changes calculated based on the selected T1, T2 and T3 comparisons in the checkbox group. Here the user can visualize the evolution of

Exp and Sm for all the proteins present in the dataset, selected proteins also appear in red on the plots.

12) The plots can be exported as .pdf and .svg, the source data of the plots can also be downloaded as .tsv.

13) Sankey diagram representing the evolution of protein trajectories along T1 and T2 based on the two-dimensional plots shown in step 12, trajectories of selected proteins are also displayed with their respective quadrant colors. Quadrants are defined in step 12 and by the selection of the Fisher score cutoff in step 3 that represents quadrant E.

14) Bar plots of Exp and Sm fold change for the selected proteins. Each condition is compared to the chosen denominator in T1 (step 2). The p-values are calculated using a paired two-tailed t-test. 15) This step and the followings are displayed on the Sankey data window (step 9). In step 15, the user can export the Sankey data of the whole dataset as presented in this window as .csv or excel formats.

16) The user can search and subset for specific proteins or features (trajectories, number of peptides) in the dataset.

17) Sankey data of the whole dataset or subset of the data selected in step 16. The data are presented with the identifiers specified in the submitted dataset. T1, T2 and T3 columns represent the quadrant in which the protein is present in each respective two-dimensional plot of Exp and Sm FC (step 11). The transition column represents the trajectory of the protein between T1 and T2 and visualized on the Sankey diagram in step 13.

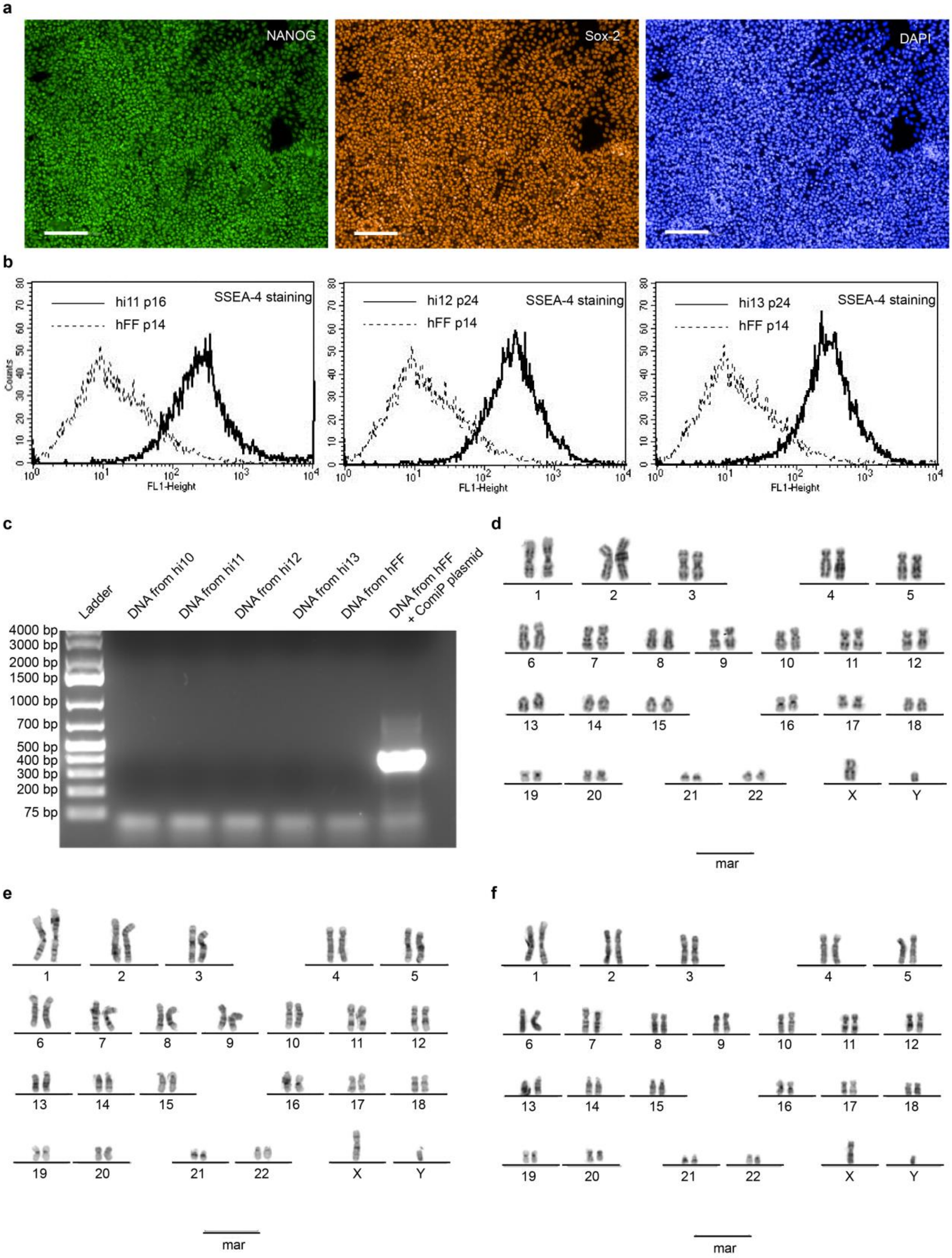

Supplementary Figure 2. Expression of pluripotency markers and genetic analysis of the iPSCs. (**a**) Immunostaining of hi12 cells with anti-Nanog (NANOG) (green), anti-Sox2 (Sox-2) (orange) antibodies and DAPI (blue) at passage 10. Scale bars, 200 µm. The experiment

was performed once. (**b**) FACS analysis of hi11, hi12 and hi13 iPSC lines for SSEA4 (markers of pluripotency) against that of hFFs. (**c**) PCR analysis of total genomic DNA isolated from hi10 (not used in this study), hi11, hi12, hi13 cells, hFFs and genomic DNA from hFF mixed with CoMiP 4in1 without shRNA p53 plasmid that was used for the reprograming (positive control). Primers 5'-GGCAGAAGGGCAAGAGAAG-3' and 5'-CTCCCGCCATCTGTTGTTAG-3' were used to detect the presence of the CoMiP sequence in the DNA preparations. The experiment was performed once. (**d**), (**e**) and (**f**) Karyotyping of hi11, hi12 and hi13 cells, respectively. No chromosomal abnormalities were observed.

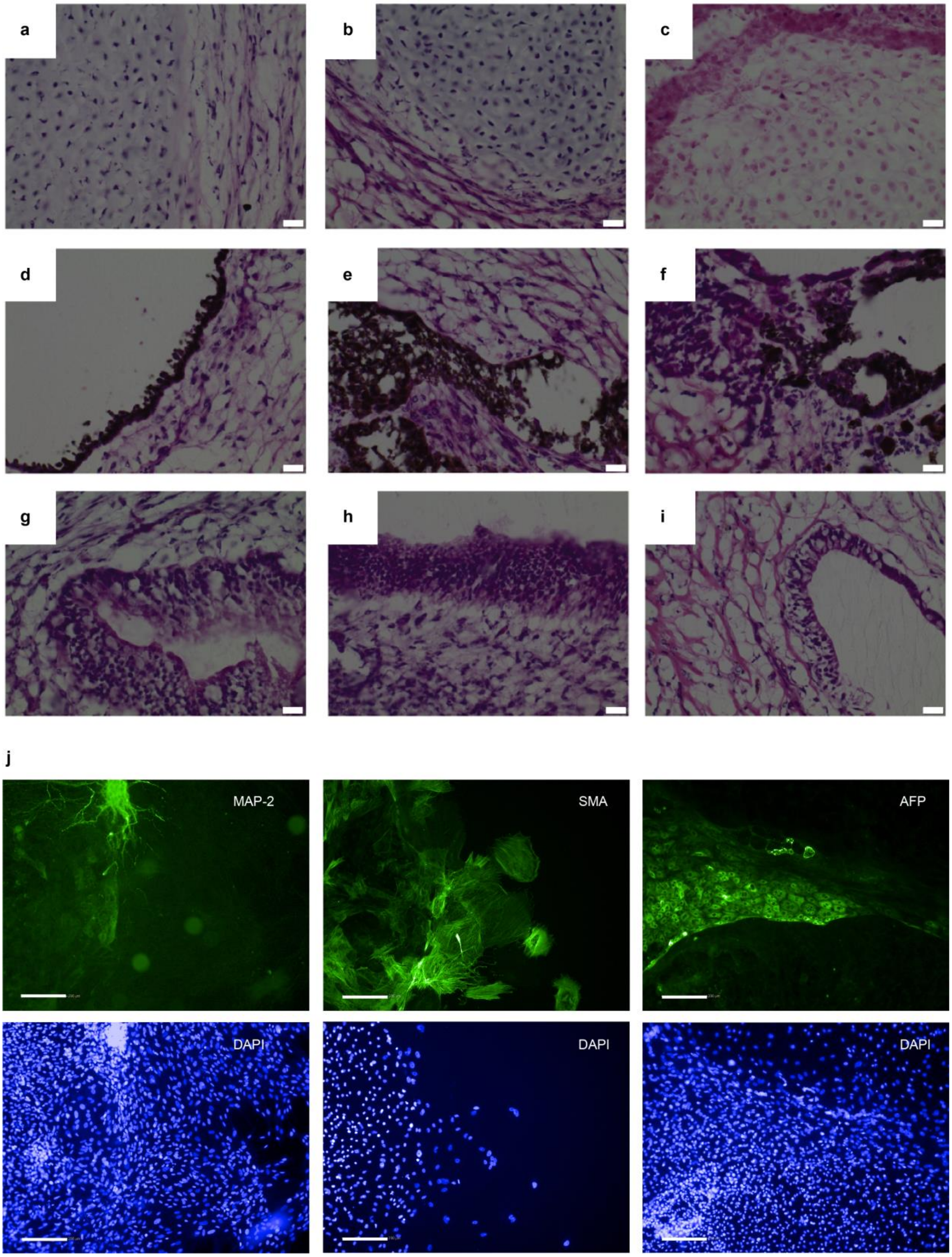

Supplementary Figure 3. Pluripotency of hi11, hi12 and hi13 cells. (**a**), (**b**) and (**c**) shows Cartilage representing formation of mesodermal germ layer in teratomas produced from hi11, hi12 and hi13 iPSC lines, respectively. (**d**), (**e**) and (**f**) shows neural cell formation

representing ectodermal germ layer in teratomas produced from hi11, hi12 and hi13 cells, respectively. (**g**), (**h**) and (**i**) shows Cylinder epithelium formation representing endodermal germ layer in teratomas produced from hi11, hi12 and hi13 cells, respectively. Scale bars, 20 µm. (**j**) Immunostaining of embryoid bodies formed from hi12 cells after 13 passages revealed expression of markers for the three embryonic germ layers: MAP-2 (green), smooth-muscle (SM) actin (green) and AFP (green). DAPI staining is shown in blue. Scale bars, 200 µm. All these experiments were performed once.

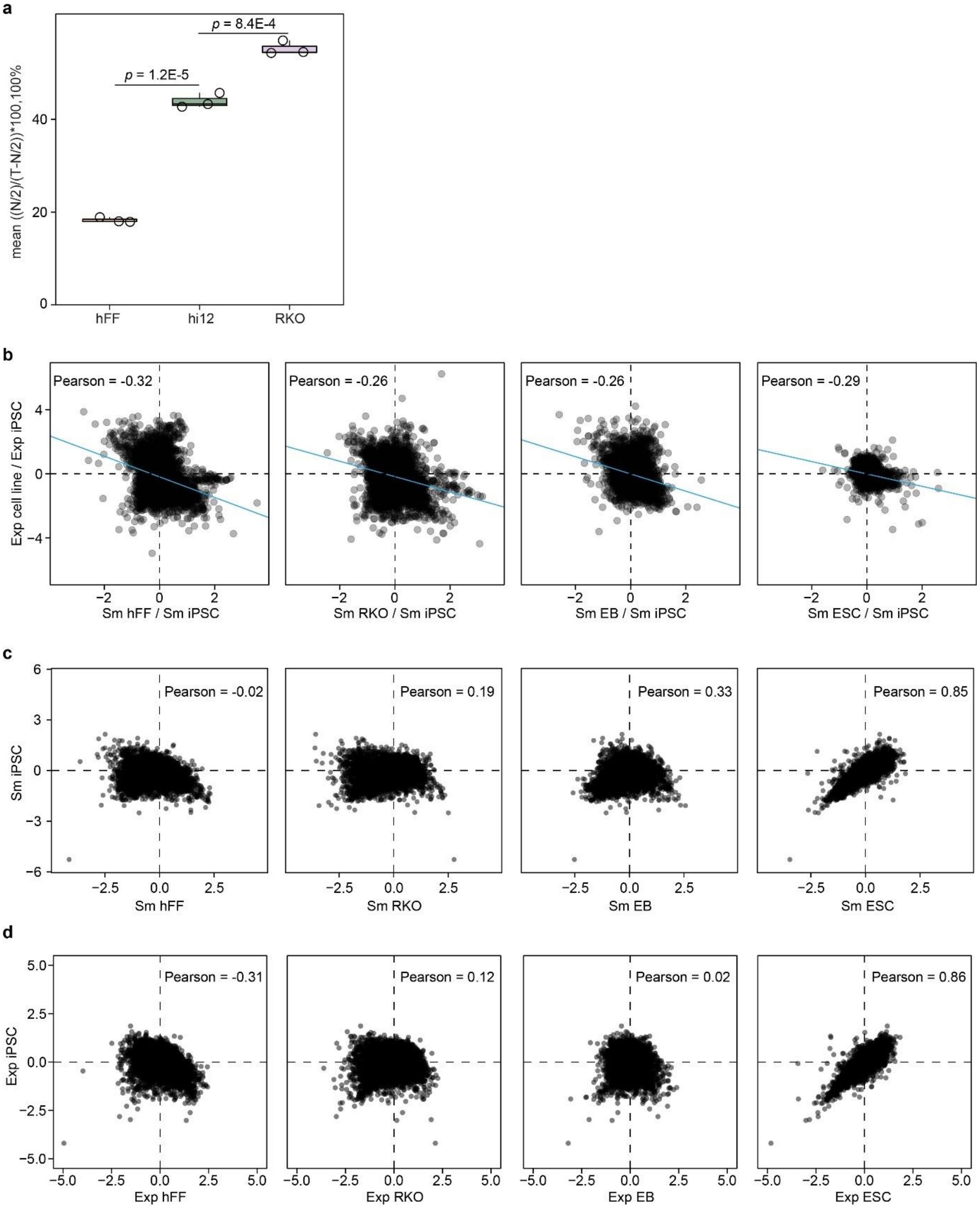

Supplementary Figure 4. Protein thermal stability and expression across cell lines compared to iPSC. (**a**) EdU incorporation in hi12 iPSCs (green), hFF (orange) and RKO (purple). T represents the number of DAPI-positive cells, N represents the number of EdU-positive cells. The data are presented as the mean percentage of EdU-positive cells in n=3

biologically independent samples. Horizonal line in the box plots represent the median, 25th and 75th percentiles and whiskers represent measurements to the 5th and 95th percentiles. P-values were calculated using a two-sided Student *t*-test, no adjustment for multiple comparisons was performed. $p < 0.05$ were considered as significant. (**b**) 2-dimensional plots of Sm FC against Exp FC of each cell line against iPSC including the regression line and Pearson correlation of Sm FC against Exp FC. (**c**) Pearson correlation of Sm in hFF, RKO, EB and ESC against iPSC. (**d**) Pearson correlation of Exp in hFF, RKO, EB and ESC against iPSC. n=3 biologically independent samples for all analysis. Source data are provided as a Source Data file.

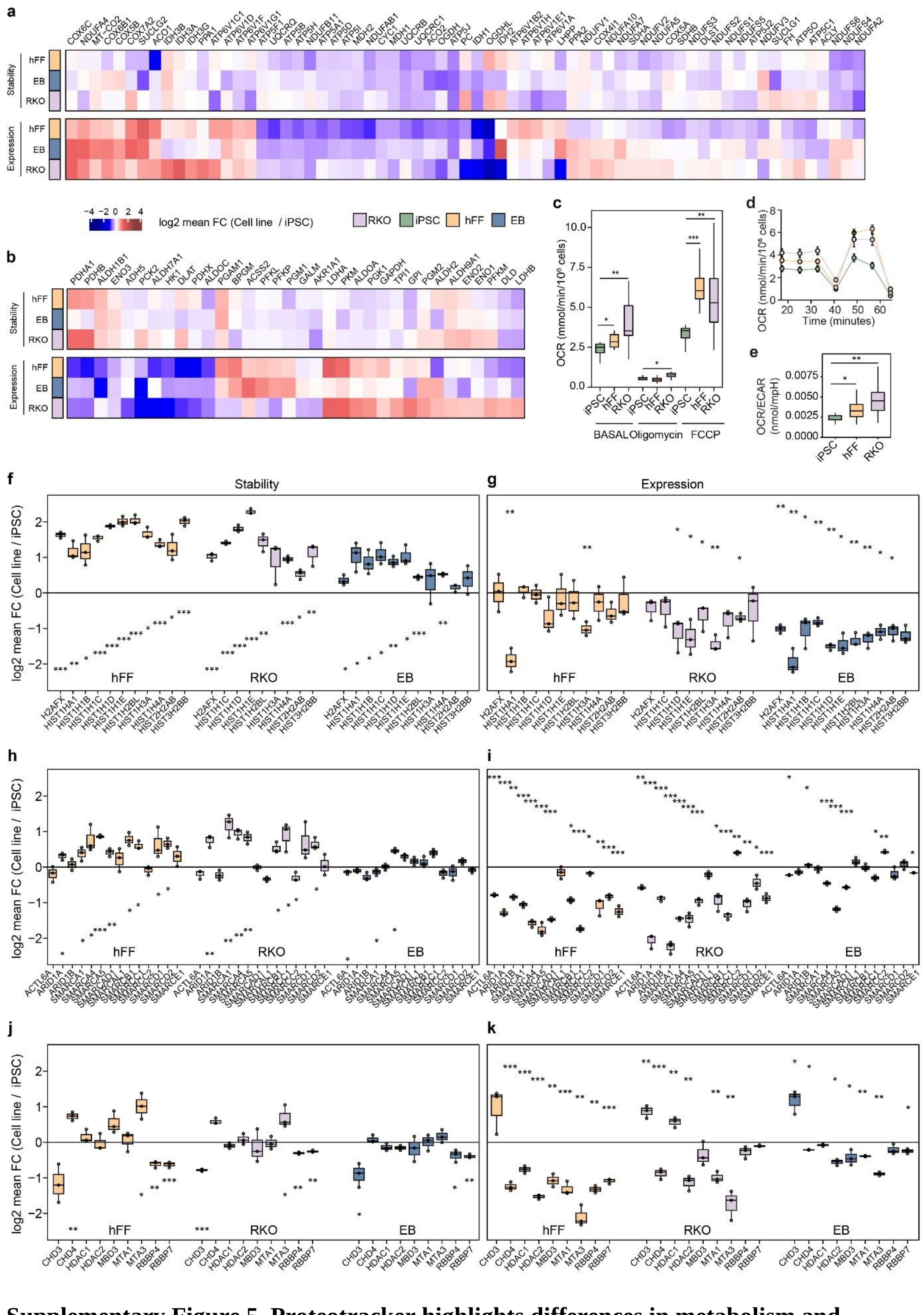

Supplementary Figure 5. Proteotracker highlights differences in metabolism and activity of chromatin remodeling complexes in PSCs compared to differentiated cells. (a)

Heatmap of Exp and Sm FCs of enzymes involved in oxidative phosphorylation in hFF, RKO and EB against iPSC. (**b**) Heatmap of mean Exp and Sm FCs of enzymes involved in glycolysis in hFF, RKO and EB against iPSC. (**c**) Oxygen consumption rate of iPSCs, hFFs and RKO cells measured using flux analysis (Seahorse). n=12 biologically independent samples for iPSC, n=14 biologically independent samples for hFF (*p*=2.0E-2), n=13 biologically independent samples for RKO (*p*=1.5E-3) at basal level; n=11 biologically independent samples for iPSC, n=13 biologically independent samples for hFF (*p*=6.9E-1), n=13 biologically independent samples for RKO (*p*=3.2E-2) after treatment with oligomycin; n=12 biologically independent samples for iPSC, n=14 biologically independent samples for hFF (*p*=2.0E-7), n=13 biologically independent samples for RKO (*p*=3.9E-3) after treatment with FCCP. Grubb's test was used to remove outliers among biological replicates. (**d**) Representative experiment of oxygen consumption rate (OCR) recording of iPSC, hFF and RKO cells using XF24 Flux Analyzer normalized on cell number. OCR was recorded at baseline (first 3 represented points) and after injections of oligomycin (1µM) at 40 min, FCCP (1-2µM) at 50 min and rotenone-antimycin (1µM) at 60 min of the experiment. Data are presented as mean ± SEM. n=7 biologically independent samples for iPSC, n=7 biologically independent samples for hFF and n=6 biologically independent samples for RKO. Grubb's test was used to remove outliers among biological replicates. (**e**) OCR/ extracellular acidification rate (ECAR) ratio during steady-state (basal levels) in iPSC, hFF and RKO. n=11 biologically independent samples for iPSC, n=14 biologically independent samples for hFF (*p*=1.8E-2) and n=12 biologically independent samples for RKO (*p*=1.1E-3). Grubb's test was used to remove outliers among biological replicates. (**f**) Thermal stability and (**g**) expression of histones in hFF, RKO and EB compared to hi12 iPSC. Proteins with inconsistent quantification between replicates were excluded from the analysis. (**h**) Thermal stability and expression (**i**) of proteins from the SWi/SNF complex in hFF, RKO and EB

respectively, against hi12 iPSCs. (**j**) Thermal stability and expression (**k**) of proteins members of the Mi2/NuRD complex in hFF, RKO and EB respectively, against iPSC. n=3 biologically independent samples when not specified. Horizonal line in the box plots represent the median, 25th and 75th percentiles and whiskers represent measurements to the 5th and 95th percentiles. Data are shown as mean ± SEM and statistical significance was calculated using a two-sided Student *t*-test against iPSC. *$p<0.05$, **$p<0.005$, ***$p<0.0005$, $p < 0.05$ were considered as significant. All replicates were biological replicates. Source data are provided as a Source Data file.

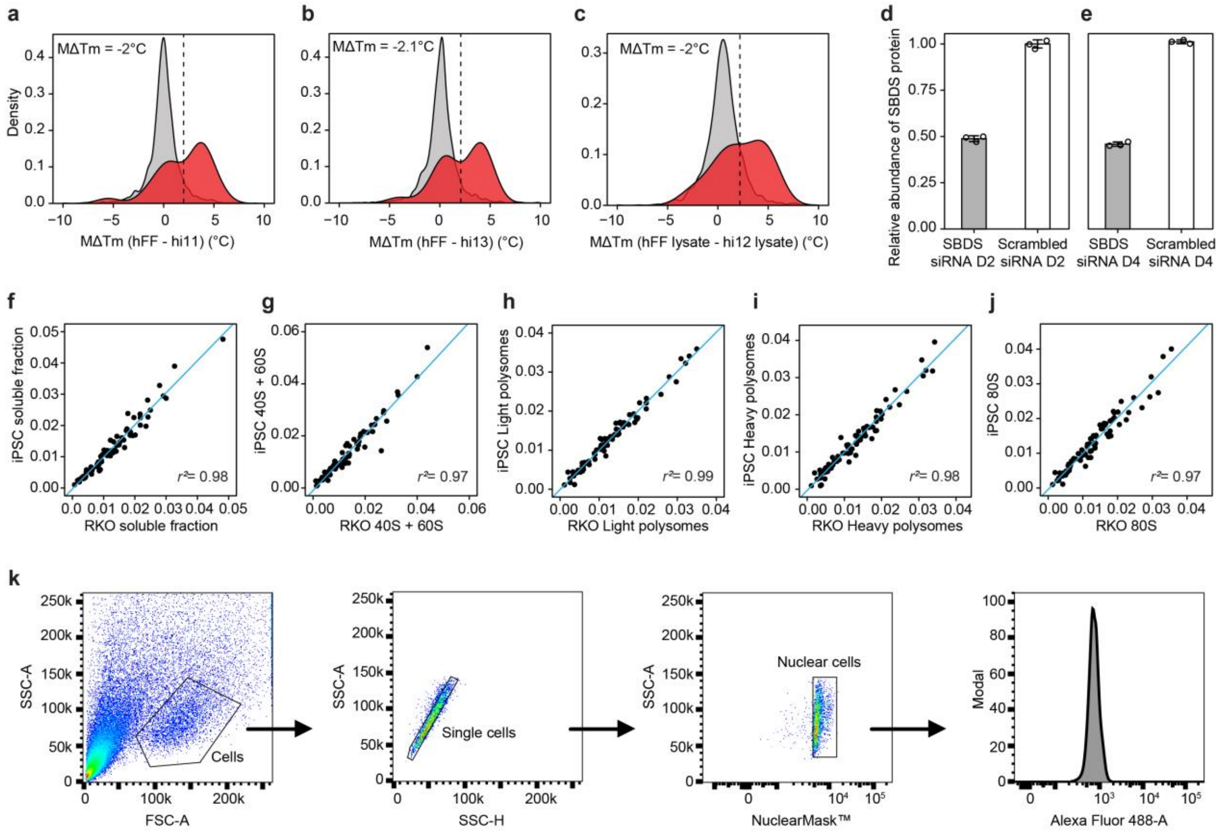

Supplementary Figure 6. Comparison of iPSCs and somatic cells; analysis of SBDS knock-down. Density distribution of the mean ΔTm of ribosomal proteins (in red) and all other proteins (in grey) of (**a**) hi11 against hFF in living cells, (**b**) hi13 against hFF in living cells, (**c**) hi12 against hFF in cell lysates (n=2 biologically independent samples).

Relative abundance of SBDS protein in hFF treated with SBDS siRNA (grey) and scrambled siRNA (white), (**d**) two days after the treatment and (**e**) four days after the treatment (n=3 biologically independent samples). Data were normalized on the mean abundance of SBDS in the cells treated with scrambled siRNA and error bars represent the standard deviation of the mean.

(**f-j**) Mean relative abundance of ribosomal proteins detected in various fractions of the ribosome density profile of hi12 iPSCs lysates versus RKO cell lysates. The individual panels correspond to (**f**) soluble protein fraction, (**g**) 40S + 60S ribosome subunits, (**h**) 80S ribosomes, (**i**) light polysomes and (**j**) heavy polysomes (n=3 biologically independent samples). The blue line represents a regression of the mean relative abundance of ribosomal

proteins between the two cell lines. (**k**) Gating strategy of the flow cytometry analysis used in Fig. 4c. Source data are provided as a Source Data file.

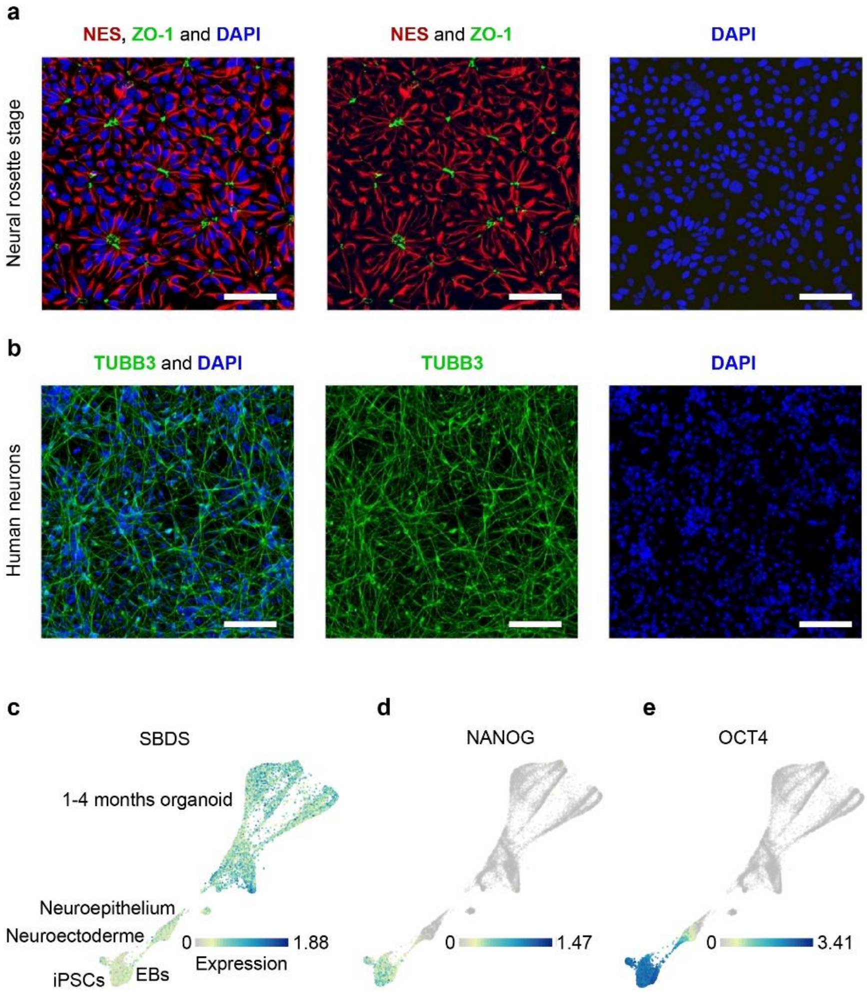

Supplementary Figure 7. Generation of human neurons from hi12 line and expression of SBDS, NANOG and OCT4 in neuronal lineage. (**a**) Immunofluorescence of neural progenitor cells after 10 days, forming the typical neural rosette structure, positive for Nestin (NES) (red) and the apical marker ZO-1 (green) in the center of each rosette, DAPI nuclear stain is in blue. Scale bars, 100 µm. The experiment was performed once. (**b**) After 12 days, most of the cells were showing neural elongated morphology with axons and neurites, typical of neuronal identity, and were positive for the neuronal marker TUBB3 (beta-III tubulin) (green). The experiment was performed once. (**c**), (**d**) and (**e**) Single-cell mRNA expression levels of SBDS, NANOG and OCT4 in human ESC (line H9) and human iPSCs (line 409b2) differentiated into cerebral organoid visualized as described in[1].

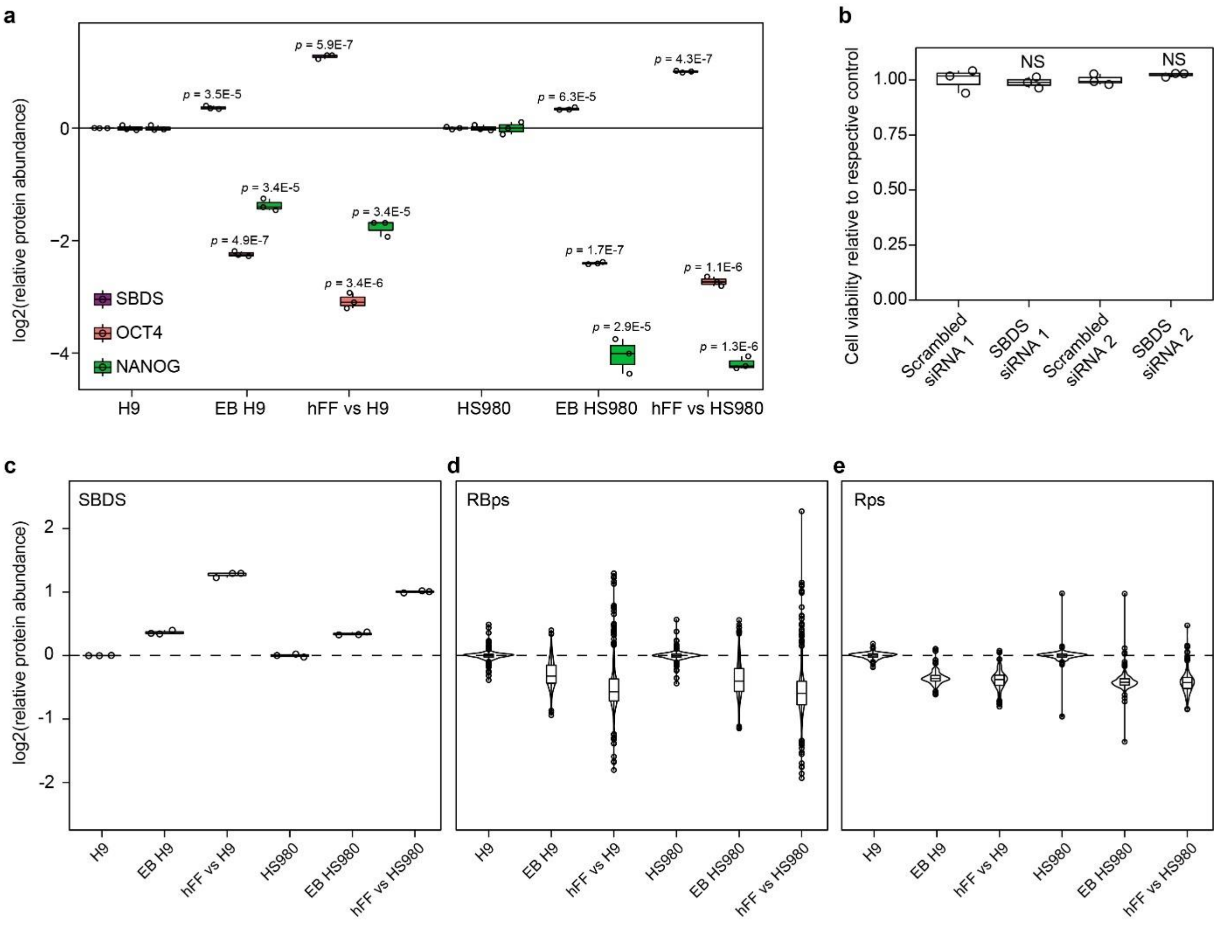

Supplementary Figure 8. Expression of SBDS, pluripotency markers, ribosome biogenesis and ribosomal proteins in EBs and hFF compared to H9 and HS980 ESCs; hi12 iPSCs viability assay after 48 h of treatment with SBDS siRNAs. (**a**) Relative protein abundances of EBs differentiated from H9 and HS980 and hFF compared to H9 and HS980. (**b**) Box plots showing cell viability upon scrambled (control = C1 and C2) and SBDS (S1 and S2) siRNA treatment normalized by the respective siRNA control C1 and S1 by C1; C2 and S2 by C2. Expression of SBDS protein (**c**), proteins involved in ribosome biogenesis (RBps) (**d**) and ribosomal proteins (Rps) (**e**), in EBs differentiated from H9 and HS980 and hFF compared to H9 and HS980. RBps encompass 231 unique proteins and Rps 78. Error bars represent ± the standard deviation of the mean. Violin plots represent the distribution of protein abundances relative to the corresponding ESC control (H9 or HS980). Horizonal line in the box plots represent the median, 25th and 75th percentiles and whiskers represent

measurements to the 5th and 95th percentiles. P-value were calculated using a two-sided Student *t*-test. $p < 0.05$ were considered as significant. n=3 biologically independent samples for all experiments. Source data are provided as a Source Data file.

Supplementary Table 1. siRNAs and primers used in the study.

Primer/siRNA	Company	Catalog number/ID	Sequence
Hs_SBDS_5 FlexiTube siRNA (SBDS siRNA 1)	Qiagen	SI03246390	5'-TTGGAAGTACTCAATCTGAAA-3' sequence with 5'-GGAAGUACUCAAUCUGAAATT-3' (sense) and 5'-UUUCAGAUUGAGUACUUCCAA-3' (antisense)
AllStar Negative Control siRNA (Scrambled siRNA 1)	Qiagen	SI03650318	
Silencer™ Select Negative Control No. 1 (Scrambled siRNA 1)	Ambion™	4390843	
Silencer® Select siRNA number s27482 (SBDS siRNA 2)	Ambion™	4392420	5'-CGAAAUCGCCUGCUACAAA-3' sequence with 5'-CGAAAUCGCCUGCUACAAATT -3' (sense) and 5'-UUUGUAGCAGGCGAUUUCGAA-3' (antisense)
NANOG TaqMan® Gene Expression Assay	ThermoFischer Scientific	Hs02387400_g1	
GAPDH TaqMan® Gene Expression Assay	ThermoFischer Scientific	Hs99999905_m1	
SOX7 TaqMan® Gene Expression Assay	ThermoFischer Scientific	Hs00846731_s1	
SOX17 TaqMan® Gene Expression Assay	ThermoFischer Scientific	Hs00751752_s1	
PAX6 TaqMan® Gene Expression Assay	ThermoFischer Scientific	Hs01088114_m1	
GATA4 TaqMan® Gene Expression Assay	ThermoFischer Scientific	Hs00171403_m1	
SBDS TaqMan® Gene Expression Assay	ThermoFischer Scientific	Hs04188846_m1	
POU5F1 (Oct4) TaqMan® Gene Expression Assay	ThermoFischer Scientific	Hs00999632_g1	

Supplementary Table 2. Proteomics experiments and LC-MS/MS systems used.

Experiment number	Experiment description	Number of replicates	Total number of TMT sets	Mass spectrometer	LC-system	Fractionation system	Number of fractions per sample
**1**	PISA of hi12, ESC, hFF, RKO and EB	3	3	Lumos Fusion Orbitrap (Thermo Fischer Scientific)	UltiMate ™ 3000 RSLCnano System (Thermo Fischer Scientific)	Ultimate™ 3000 RSLCnano System (Dionex)	24
**2**	TPP of hi12 against hFF in cells	2	4	Fusion Orbitrap (Thermo Fischer Scientific)	EASY-nLC 1000 (Thermo Fischer Scientific)	Pierce™ High pH Reversed-Phase Peptide Fractionation Kit (Thermo Fischer Scientific)	8
**3**	TPP of hi12 against RKO in cells	2	4	Elite Orbitrap (Thermo Fischer Scientific)	Same as above.	Same as above.	8
**4**	TPP of hi11 and hi13 against hFF in cells	2	6	Q-Exactive plus Orbitrap (Thermo Fischer Scientific)	Same as above.	Same as above.	8
**5**	TPP of hi12 against hFF in lysate	2	4	Fusion Orbitrap (Thermo Fischer Scientific)	Same as above.	Same as above.	8
**6**	Analysis of sucrose density gradient fractions	3	3	Elite Orbitrap (Thermo Fischer Scientific)	UltiMate ™ 3000 RSLCnano System (Thermo Fischer Scientific)	None.	/
**7**	Expression proteomics of hi12 against hFF and EBs	3	1	Same as above.	Same as above.	Ultimate™ 3000 RSLCnano System (Dionex)	24
**8**	Expression proteomics of hi12 against RKO and HT29	3	1	Same as above.	Same as above.	Same as above.	24
**9**	Expression proteomics of hi12 against neurons	3	1	Same as above.	Same as above.	Same as above.	24
**10**	SBDS siRNA KD 2 days treatment and 4 days treatment in hFF	3	2	Q-Exactive Orbitrap (Thermo Fischer Scientific)	EASY-nLC 1000 (Thermo Fischer Scientific)	Pierce™ High pH Reversed-Phase Peptide Fractionation Kit (Thermo Fischer Scientific)	8
**11**	Time-serie of EBs induction and SBDS siRNA KD	3	3	Q-Exactive HF Orbitrap (Thermo Fischer Scientific)	UltiMate ™ 3000 RSLCnano System (Thermo Fischer Scientific)	Ultimate™ 3000 RSLCnano System (Dionex)	24
**12**	Expression proteomics of H9, EBs and hFF	3	2	Q-Exactive HF Orbitrap (Thermo Fischer Scientific)	UltiMate ™ 3000 RSLCnano System (Thermo Fischer Scientific)	Ultimate™ 3000 RSLCnano System (Dionex)	24

Supplementary Table 3. LC-MS/MS parameters.

Experiment	Gradient duration (min)	TopN	MS resolution	MS AGC target	MS max injection time (ms)	Scan range (m/z)	HCD energy (%)	MS/MS resolution	MS/MS AGC target	MS/MS max injection time	Isolation window (Da)	Fixed first mass (m/z)	Dynamic exclusion (s)
**1**	120	Not applicable	120000	Standard	Auto	400-1600	35	60000	250 %	Auto	1.6	100	60
**2**<br>**5**	180<br>140	20<br>-	120000<br>-	1E6<br>-	50<br>-	400-1600<br>-	40<br>-	60 000<br>-	1E5<br>-	105<br>-	0.7<br>-	105<br>-	60<br>-
**3**<br>**6**<br>**7,8,9**	120<br>210<br>110	10<br>-<br>-	120000<br>-<br>-	1E6<br>-<br>-	200<br>-<br>-	375-1500<br>-<br>-	35<br>-<br>-	30000<br>-<br>-	5E4<br>-<br>-	200<br>-<br>-	1.6<br>-<br>-	100<br>-<br>-	60<br>-<br>-
**4**	120	10	70000	3E6	250	375-1400	35	35000	2E5	120	1.2	100	15
**10**	140	15	70000	1E6	120	375-1500	32	70000	2E5	120	1.2	100	45
**11**	120	17	120000	3E6	100	375-1500	33	60000	2E5	120	1.6	100	45
**12**	120	20	120000	5E6	100	375-1500	33	45000	2E5	93	1.6	100	60

Supplementary References

1. Kanton, S. *et al.* Organoid single-cell genomic atlas uncovers human-specific features of brain development. *Nature* **574**, 418–422 (2019).
