# Supplementary material for: An integrative proteomics method identifies a regulator of translation during stem cell maintenance and differentiation
Source: Nat Commun. 2021 Nov 12;12:6558. doi: 10.1038/s41467-021-26879-4 (PMC8590018; doi:10.1038/s41467-021-26879-4)
Supplement: Supplementary file 3 — Description of Additional Supplementary Files [file 41467_2021_26879_MOESM3_ESM.docx]

**Description of Additional Supplementary Files**

**File Name:** Supplementary Data 1

**Description:** PISA-Express data. Sm and Exp parameters for each protein passing our quality control in iPSC hi12, H9, hFF, RKO and EBs differentiated from hi12, n=3.

**File Name:** Supplementary Data 2

**Description:** Proteomics expression data. Table 2-1: Expression of ribosomal proteins from sucrose density gradient fractionation of RKO and iPSC hi12. Table 2-2: Protein expression in hFF, HT29, RKO, neurons, EBs normalized by hi12. Table 2-3: Normalized protein expression data of SBDS siRNA and scrambled siRNA knock down at day 2 and day 4 of treatment, n=3. Table 2-4: Normalized protein expression in time-series of EB development differentiated from iPSC hi12 at day 0 (iPSC hi12), day 3, day 6 and day 9 and SBDS and scrambled siRNA treatment of EBs as well as mean ratio of EB versus iPSCs and SBDS versus scrambled siRNA, n=3. Table 2-5: Normalized protein expression in H9, EBs differentiated from H9 and H980 and EBs differentiated from H980, hFF was added as a reference in both experiment, n=3.

**File Name:** Supplementary Data 3

**Description:** Protein melting points in thermal proteome profiling experiments. Table 3-1: Tm and ΔTm of hFF – iPSC hi12 n=2. Table 3-2: Tm and ΔTm of RKO – iPSC hi12 n=2. Table 3-3: Tm and ΔTm of hFF – iPSC hi11 n=2. Table 3-4: Tm and ΔTm of hFF – iPSC hi13 n=2. Table 3- 5: Tm and ΔTm of hFF – iPSC hi12 in cell lysate n=2.

**File Name:** Supplementary Data 4

**Description:** Microarray mRNA analysis of hi12 and hFF. n=3.
