# Supplementary material for: An integrative proteomics method identifies a regulator of translation during stem cell maintenance and differentiation
Source: Nat Commun. 2021 Nov 12;12:6558. doi: 10.1038/s41467-021-26879-4 (PMC8590018; doi:10.1038/s41467-021-26879-4)
Supplement: Supplementary file 8 — Reporting Summary [file 41467_2021_26879_MOESM8_ESM.pdf]

nature portfolio

Corresponding author(s): Roman Zubarev
Last updated by author(s): Oct 21, 2021

Reporting Summary

Nature Portfolio wishes to improve the reproducibility of the work that we publish. This form provides structure for consistency and transparency in reporting. For further information on Nature Portfolio policies, see our Editorial Policies and the Editorial Policy Checklist.

Statistics

For all statistical analyses, confirm that the following items are present in the figure legend, table legend, main text, or Methods section.

n/a	Confirmed	
☐	☒	The exact sample size (*n*) for each experimental group/condition, given as a discrete number and unit of measurement
☐	☒	A statement on whether measurements were taken from distinct samples or whether the same sample was measured repeatedly
☐	☒	The statistical test(s) used AND whether they are one- or two-sided<br>*Only common tests should be described solely by name; describe more complex techniques in the Methods section.*
☒	☐	A description of all covariates tested
☒	☐	A description of any assumptions or corrections, such as tests of normality and adjustment for multiple comparisons
☐	☒	A full description of the statistical parameters including central tendency (e.g. means) or other basic estimates (e.g. regression coefficient) AND variation (e.g. standard deviation) or associated estimates of uncertainty (e.g. confidence intervals)
☐	☒	For null hypothesis testing, the test statistic (e.g. *F*, *t*, *r*) with confidence intervals, effect sizes, degrees of freedom and *P* value noted<br>*Give P values as exact values whenever suitable.*
☒	☐	For Bayesian analysis, information on the choice of priors and Markov chain Monte Carlo settings
☒	☐	For hierarchical and complex designs, identification of the appropriate level for tests and full reporting of outcomes
☐	☒	Estimates of effect sizes (e.g. Cohen's *d*, Pearson's *r*), indicating how they were calculated

Our web collection on statistics for biologists contains articles on many of the points above.

Software and code

Policy information about availability of computer code

Data collection	Thermo Xcalibur 4.0, Brandel Peak Chart recording software version 2.08, CFX manager version 3.0, CellQuest, DIVA 8.0.1, Transcriptome Analysis Console 3.0.
Data analysis	R version 3.6.1., MaxQuant 1.6.2.3, Flowjo 8.8.6, Gene Ontology enRIchment anaLysis and visuaLizAtion tool (GOrilla), Excel 2016, TPP R package version 3.13, DAVID version 6.8, CFX manager version 3.0.<br>The code for the ProteoTracker web-interface is available in GitHub (https://github.com/RZlab/ProteoTracker) and can be cited with DOI: 10.5281/zenodo.5549677.

For manuscripts utilizing custom algorithms or software that are central to the research but not yet described in published literature, software must be made available to editors and reviewers. We strongly encourage code deposition in a community repository (e.g. GitHub). See the Nature Portfolio guidelines for submitting code & software for further information.

Data

Policy information about availability of data

All manuscripts must include a data availability statement. This statement should provide the following information, where applicable:

- Accession codes, unique identifiers, or web links for publicly available datasets
- A description of any restrictions on data availability
- For clinical datasets or third party data, please ensure that the statement adheres to our policy

The authors declare that there are no restrictions on data availability and that all the data supporting the findings of this article are available within the article and Supplementary information files.
All relevant data are available from the corresponding authors (S. R. and R. A. Z.).

The mass spectrometry proteomics data have been deposited to ProteomeXchange Consortium (http://proteomecentral.proteomexchange.org) via the PRIDE partner repository with data set identifier PXD018453 [http://www.ebi.ac.uk/pride/archive/projects/PXD018453] (PISA-Express data), PXD014830 [http://www.ebi.ac.uk/pride/archive/projects/PXD014830] (TPP data of hi11, hi12, hi13, hFF, RKO in cells; TPP data of hi12 and hFF in cell lysates; ribosomal protein expression data in hi12 and RKO; protein expression data in hFF, hi12, HT29, Neurons, EBs, RKO; SBDS KD protein expression data in hFF; SBDS KD protein expression data after EB induction in hi12) and PXD015874 [https://www.ebi.ac.uk/pride/archive/projects/PXD015874] (Protein expression data of EB induction in H9 and HS980).
The microarray RNA analysis data were deposited to GEO repository under the accession number GSE135409 [https://0-www-ncbi-nlm-nih-gov.brum.beds.ac.uk/geo/query/acc.cgi?acc=GSE135409].
Statistics from the TPP data generated by the TPP R package version 3.13 from Franken et al.10 are deposited in the Zenodo repository with DOI: https://doi.org/10.5281/zenodo.5018241. The ProteoTracker tool is freely available at http://www.proteotracker.genexplain.com.
Proteomics data were searched against the UniProt human database 2019_05 excluding protein isoforms (73 910 entries).
Source data for every plot are provided with this paper.

Field-specific reporting

Please select the one below that is the best fit for your research. If you are not sure, read the appropriate sections before making your selection.

☒ Life sciences ☐ Behavioural & social sciences ☐ Ecological, evolutionary & environmental sciences

For a reference copy of the document with all sections, see nature.com/documents/nr-reporting-summary-flat.pdf

Life sciences study design

All studies must disclose on these points even when the disclosure is negative.

Sample size	No Statistical methods were used to predetermine sample size. Biological duplicates were used for thermal proteome profilling analysis and biological triplicates (or in some cases more replicates) have been used for all other experiments in the study to gain appropriate power for discovery of true positive events. The sample sizes were chosen based on similar studies published previously.
Data exclusions	For LC-MS/MS data, only proteins identified with two peptides or more and without missing values in any of the samples and replicates were considered for statistical analysis.<br>The rationale behind selecting proteins with two peptides or more is that protein quantification is unreliable with one peptide only, this is the gold standard in the proteomics field.<br>We chose to exclude proteins with missing values due to the stochastic precursors selection of data-dependent acquisition, which is common practice in proteomics.
Replication	All experiments were performed in at least two independent biological replicates as detailed in the paper. TPP data recorded with iPSC line hi12 were also confirmed with 2 other iPSCs lines (hi11 and hi13) reprogrammed from the same parental hFF line. SBDS expression levels in hFF and hi12 were confirmed by RNA microarray analysis. SBDS and pluripotency markers LC-MS/MS data were corroborated in 2 separate cell lines and were also confirmed by qRT-PCR. SBDS knock down were confirmed in 3 separate experiments, and SBDS knock in in 2 separate experiments. Ribosome profiles observations were confirmed by protein synthesis rate measurements.<br>Conclusions were drawn from reproducible effects in all replicates of the datasets.<br>All attempts at replication were successful.
Randomization	This is not relevant in this study, as we used biological replicates from cell lines or cell lysates with no covariates such as animal to animal or patient to patient variability.
Blinding	Blinding was not performed in this study. This is not relevant to our study since the data collected were analyzed using unbiased statistics and no covariates exists that could introduce a certain bias in the analysis.

Reporting for specific materials, systems and methods

We require information from authors about some types of materials, experimental systems and methods used in many studies. Here, indicate whether each material, system or method listed is relevant to your study. If you are not sure if a list item applies to your research, read the appropriate section before selecting a response.

Materials & experimental systems

n/a	Involved in the study
☐	☒ Antibodies
☐	☒ Eukaryotic cell lines
☒	☐ Palaeontology and archaeology
☐	☒ Animals and other organisms
☒	☐ Human research participants
☒	☐ Clinical data
☒	☐ Dual use research of concern

Methods

n/a	Involved in the study
☒	☐ ChIP-seq
☐	☒ Flow cytometry
☒	☐ MRI-based neuroimaging

Antibodies

Antibodies used	SSEA4 Monoclonal Antibody (Invitrogen), catalogue # MA1-021-D488, lot # TH274753. Dilution 1:200.<br>AFP (R@D Systems), catalogue # MAB1368, lot # HPH0209081. Dilution 1:100.<br>Nanog (R@D Systems), catalogue # AF1997, lot # KKJ0409101. Dilution 1:10.<br>SMA (Merck-Millipore), catalogue # A5228. Dilution 1:250.<br>MAP-2 (Millipore), catalogue # MAB3418, lot # LV 1796719. Dilution 1:200.<br>Sox2 (R@D Systems), catalogue # MAB2018, lot # KGQ020841. Dilution 1:10.<br>rabbit anti-Nestin (Atlas Antibodies), catalogue # HPA007007. Dilution 1:500.<br>mouse monoclonal anti-ZO-1 (Molecular Probes), catalogue # 339194. Dilution 1:500.<br>mouse monoclonal anti-βIII Tubulin (TUBB3)(Promega), catalogue # G7121. Dilution 1:500.
Validation	-SSEA4 antibody staining was validated by the manufacturer for flow cytometry analysis. According to manufacturer: MA1-021-D488 has been successfully used in ICC/IF and flow cytometry applications on human samples.<br>-AFP antibody staining was validated by the manufacturer for immunocytochemistry. Alpha-Fetoprotein AFP was detected in immersion fixed HepG2 human hepatocellular carcinoma cell line.<br>-Nanog antibody staining was validated by the manufacturer for immunocytochemistry. Nanog was detected in immersion fixed BG01V human embryonic stem cells.<br>-SMA antibody staining was validated by the manufacturer for immunocytochemistry and published. (Shen, Yc., Shami, A.N., Moritz, L. et al. TCF21+ mesenchymal cells contribute to testis somatic cell development, homeostasis, and regeneration in mice. Nat Commun 12, 3876 (2021). https://doi.org/10.1038/s41467-021-24130-8)<br>-Anti-MAP2 Antibody was validated by the manufacturer to detect levels of MAP2 and has been published and validated for use in immunohistochemistry and Western Blot. (Interruption of beta-catenin signaling reduces neurogenesis in Alzheimer's disease. He, P; Shen, Y. The Journal of neuroscience : the official journal of the Society for Neuroscience 29 6545-57 2009) (The HIF-1/glial TIM-3 axis controls inflammation-associated brain damage under hypoxia. Koh, HS; Chang, CY; Jeon, SB; Yoon, HJ; Ahn, YH; Kim, HS; Kim, IH; Jeon, SH; Johnson, RS; Park, EJ. Nature communications 6 6340 2015).<br>-Sox2 antibody staining was validated by the manufacturer for Western Blot, immunocytochemistry and flow cytometry. Sox2 was detected in immersion fixed BG01V human embryonic stem cells.<br>-Nestin antibody was validated by the manufacturer for Western Blot and immunohistochemistry in multiple human tissues including kidney and liver. Orthogonal validation of protein expression using IHC by comparison to RNA-seq data of corresponding target in high and low expression tissues. Validation of protein expression in IHC by comparing independent antibodies targeting different epitopes of the protein. Validated against independent antibody Anti-NES HPA026111.<br>-ZO-1 antibody was validated by the manufacturer for immunocytochemistry against CaCo2 cells.<br>-anti-βIII Tubulin antibody was validated for immunohistochemistry by the manufacturer in rat cerebellum.

Eukaryotic cell lines

Policy information about cell lines

Cell line source(s)	HFF (ATCC® CRL-2429™, male)<br>RKO cells (ATCC® CRL-2577™, female)<br>HT-29 (ATCC® HTB-38™, female)<br>Wild-type H9 (WiCell WA-09, female)<br>HS980 was provided by Prof. Outi Hovatta.
Authentication	None of the cell lines were authenticated as cells were used directly from the commercial source or in the case of HS980 directly from the aliquots obtained from Prof. Outi Hovatta.
Mycoplasma contamination	All cell lines tested negative for mycoplasma.
Commonly misidentified lines<br>(See ICLAC register)	No misidentified cell lines were used in this study.

Animals and other organisms

Policy information about studies involving animals; ARRIVE guidelines recommended for reporting animal research

Laboratory animals	The study involved young (8-week-old) NOD SCID mice. The mice were males. The mice were fed and had water Ad libitum. The temperature was kept between 18 and 23 °C and the humidity was kept between 40 and 60%, and these parameters were checked daily. The mice were subjected to day/night light cycles with 14 h of light and 10 h of dark.
Wild animals	This study did not involve wild animals.
Field-collected samples	This study did not involve field-collected samples.
Ethics oversight	All procedures were approved by the Ethics Committee on Animal Experiment, Sweden (ethical approvals number S198-11 and S31-14).

Note that full information on the approval of the study protocol must also be provided in the manuscript.

Flow Cytometry

Plots

Confirm that:

☒ The axis labels state the marker and fluorochrome used (e.g. CD4-FITC).

☒ The axis scales are clearly visible. Include numbers along axes only for bottom left plot of group (a 'group' is an analysis of identical markers).

☒ All plots are contour plots with outliers or pseudocolor plots.

☒ A numerical value for number of cells or percentage (with statistics) is provided.

Methodology

Sample preparation	Protein synthesis rate in hi12, hFF and RKO was measured using Click-iT® Plus OPP Protein Synthesis Assay (Thermo Fischer Scientific). Briefly hi12, hFF and RKO were treated with OPPuro at exponential growth phase and samples were processed according to manufacturer's protocol for flow cytometry analysis. Single-cell suspension were prepared by filtering through 40 µm cell strainers (BD Falcon).
Instrument	SORP BD LSRII Analytic Flow Cytometer (BD Biosciences), serial number: H47100159.
Software	Data were acquired with DIVA software version 8.0.1 and analysed using Flowjo version 8.8.6
Cell population abundance	5000 cells positives for nuclear staining were selected for each cell line and replicate for analysis of fluorescence signal.
Gating strategy	Gating was performed on cells/singlet/NuclearMask™ Blue stain positive cells.

☒ Tick this box to confirm that a figure exemplifying the gating strategy is provided in the Supplementary Information.
